# Knowledge, Awareness and Practices Regarding Dengue Fever among the Adult Population of Dengue Hit Cosmopolitan

**DOI:** 10.1371/journal.pone.0002620

**Published:** 2008-07-09

**Authors:** Ahmed Itrat, Abdullah Khan, Sunniya Javaid, Mahwash Kamal, Hassan Khan, Sannia Javed, Saira Kalia, Adil Haleem Khan, Muhammad Imran Sethi, Imtiaz Jehan

**Affiliations:** 1 Medical College, Aga Khan University, Karachi, Pakistan; 2 Department of Community Health Sciences, Aga Khan University, Karachi, Pakistan; Institute of Human Virology, United States of America

## Abstract

**Background:**

The World health Organization (WHO) declares dengue and dengue hemorrhagic fever to be endemic in South Asia. Despite the magnitude of problem, no documented evidence exists in Pakistan which reveals the awareness and practices of the country's adult population regarding dengue fever, its spread, symptoms, treatment and prevention. This study was conducted to assess the level of knowledge, attitudes and practices regarding dengue fever in people visiting tertiary care hospitals in Karachi, Pakistan.

**Methods:**

A cross-sectional pilot study was conducted among people visiting tertiary care hospitals in Karachi. Through convenience sampling, a pre-tested and structured questionnaire was administered through a face-to-face unprompted interview with 447 visitors. Knowledge was recorded on a scale of 1–3.

**Results:**

About 89.9% of individuals interviewed had heard of dengue fever. Sufficient knowledge about dengue was found to be in 38.5% of the sample, with 66% of these in Aga Khan University Hospital and 33% in Civil Hospital Karachi. Literate individuals were relatively more well-informed about dengue fever as compared to the illiterate people (p<0.001). Knowledge based upon preventive measures was found to be predominantly focused towards prevention of mosquito bites (78.3%) rather than eradication of mosquito population (17.3%). Use of anti- mosquito spray was the most prevalent (48.1%) preventive measure. Television was considered as the most important and useful source of information on the disease.

**Conclusion:**

Adult population of Karachi has adequate knowledge related to the disease ‘dengue’ on isolated aspects, but the overall prevalence of ‘sufficient knowledge’ based on our criteria is poor. We demonstrated adequate prevalence of preventive practices against the disease. Further studies correlating the association between knowledge and its effectiveness against dengue will be helpful in demonstrating the implications of awareness campaigns.

## Introduction

Dengue virus infection is increasingly recognized as one of the world's emerging infectious diseases [1]–[4]. About 50–100 million cases of dengue fever and 500,000 cases of Dengue Hemorrhagic Fever (DHF), resulting in around 24,000 deaths, are reported annually [5], [6]. Over half of the world's population resides in areas potentially at risk for dengue transmission, making dengue one of the most important human viral disease transmitted by arthropod vectors in terms of morbidity and mortality [7].

WHO declares dengue and dengue hemorrhagic fever to be endemic in the Asian sub-continent. Presently, dengue is endemic in 112 countries of the world [1]. The only reported dengue fever outbreak in Pakistan occurred in June 1994, and a large number of cases have been reported in the region since then [8], [9].

In a developing country like Pakistan, preventable diseases such as dengue have the potential to cause the greatest mortality. Despite the magnitude of problem, no documented evidence exists on the awareness and practices of the adult population regarding dengue fever.

Given this void, the aim of our study was to assess the level of knowledge about dengue, its spread, symptoms and prevention among the population of Karachi, a city worst hit by dengue outbreaks in recent times. We were also keen to find out the level of awareness regarding the preventive measures against dengue fever

## Methods

A cross-sectional study assessing the knowledge, attitudes and practices regarding dengue was performed among people visiting the state run Civil Hospital (CHK) and Aga Khan University Hospital (AKUH), two major tertiary care facilities in Karachi, during the period November–December 2006. During this period dengue outbreak led to over 2000 hospitalizations and resulted in more than 50 deaths [9].

A sample size of 462 subjects was required to fulfill the objectives of our study at a 95% confidence level. We assumed a 50% prevalence of good knowledge and attitudes, 5% bound-of-error, and inflated the sample by 20% to account for non-respondents and incomplete interviews.

Residents of Karachi aged 18 years or above, who were visiting the outpatient departments (OPDs), either as patients or their attendants, during the periods November to December 2006, formed the sample population. Using convenience sampling, the study participants were approached and verbal consent for a face-to-face interview was sought. People were interviewed in Urdu, which is the national language of the country. People who failed to respond to all questions or who left before completing the interview were excluded. All medical personnel including doctors, nurses and medical students were excluded from the study, as well as people unable converse in Urdu.

Face-to-face interview was based on a pretested questionnaire which comprised of 32 questions, and was divided into four sections which included: 1) demographics, 2) recent experience with dengue, 3) knowledge and attitudes about the disease, and 4) practices related to prevention against dengue. Interviews were conducted by fourth year medical students who underwent training in interviewing techniques under professional supervision. To ensure reliability, the interviewers thoroughly discussed the questionnaires before collecting data.

Knowledge score was assessed on three essential questions: 1) mode of spread of dengue, 2) common symptoms of dengue, and 3) preventive measures against the disease. According to our criteria, for “sufficient knowledge”, the respondent needed to have correct responses to all the three questions. All other combinations were termed “insufficient knowledge”.

At the end of the interview each respondent was provided a handout with information relating to dengue fever. This handout contained Information on the vector, its breeding sites, biting time; dengue fever, its transmission, symptoms, treatment and preventive measures. It also briefed about the difference between dengue fever and dengue hemorrhagic fever.

Data was double entered and analyzed in Statistical Package for Social Sciences 13.0 (SPSS, Inc., Chicago, IL, USA). Results were recorded as frequencies, standard deviations (SD), and p-values. For all purposes, a p-value of <0.05 was considered as the criteria of significance.

The study was conducted in compliance with ‘Ethical Principles for Medical Research Involving Human Subjects’ of Helsinki Declaration. Confidentiality of each participant was ensured and any possible ethical concerns were discussed prior to starting the survey by the team members and the supervising faculty.

The study was approved by the Ethical Review Committee as well as the Department of Community Health Sciences Department, Aga Khan University.

## Results

About 447 (97%) of the total interviews were completed and included in the study. Out of the total study participants, 402 (89.9%) had previously heard about dengue. Majority (47.2%) belonged to the age group of 26–40 years. Considerable majority of respondents were graduates (21%). Monthly household income of the majority of people ranged between Rs. 5,000–10,000. (∼US $80–160). Table 1 describes the demographics of the study population.

**Table 1:** Basic Demographic Features.

Age (years)	Total		AKUH		CHK	
	n	%	n	%	n	%
18–25	**121**	**27.1**	58	25.8	63	28.4
26–40	**211**	**47.2**	114	50.7	97	43.7
41–64	**105**	**23.5**	47	20.9	58	26.1
65+	**10**	**2.2**	6	2.7	4	1.8
**Sex**						
Male	**283**	**63.3**	126	56	157	70.7
Female	**164**	**36.7**	99	44	65	29.3
**Marital Status**						
Single	**103**	**23.0**	55	24.4	48	21.6
Engaged	**9**	**2.0**	5	2.2	4	1.8
Married	**325**	**72.7**	161	71.6	164	73.9
Divorced	**5**	**1.1**	03	1.3	2	0.9
Widow	**5**	**1.1**	1	0.4	4	1.8
**Education**						
Illiterate	**88**	**19.7**	14	6.2	74	33.3
Read/write	**25**	**5.6**	9	4	16	7.2
Primary	**34**	**7.6**	6	2.7	28	12.6
Secondary	**37**	**8.3**	17	7.6	20	9
Grade 10	**79**	**17.7**	33	14.7	46	20.7
Intermediate	**75**	**16.8**	54	24	21	9.5
Graduate	**94**	**21.0**	78	34.7	16	7.2
Postgraduate	**15**	**3.4**	14	6.2	1	0.5
**Income** * §						
<3000	**39**	**9.3**	4	1.9	35	17.1
3000–5000	**77**	**18.4**	20	9.3	57	27.8
5000–10000	**112**	**26.7**	32	15	80	39
10000–20000	**79**	**18.9**	58	27.1	21	10.2
20000–350000	**50**	**11.9**	44	20.6	6	2.9
>35000	**62**	**14.8**	56	26.2	6	2.9

* Monthly Income in Rupees.

§ 28 people refused to comment.

Data revealing the awareness of dengue transmission, its symptoms and treatment are shown in Table 2. Majority (89.9%) of the respondents had heard about dengue, and most (84%) of them believed that the disease was infectious and transmissible. When asked about the common symptoms, fever was the most consistent response (81.5%) followed by bleeding from any site (41.9%).

**Table 2:** Awareness on Dengue Spread, Symptom and Treatment.

Variable	n	%
**Aware of dengue** *		
Yes	402	89.9
No	45	10.1
**Is Dengue Transmissible?**		
Yes	341	84.8
No	44	10.9
Don't know	17	4.2
**Mode of spread** ** **** Φ		
Mosquito bite	325	86.9
Fly bite	1	0.3
Dirty drinking water	23	6.1
Unhygienic food	12	3.2
Don't know	13	3.5
**Human to human spread?**		
Yes	89	22.1
No	240	59.7
Don't know	73	18.2
**Common symptoms** Φ		
Fever	325	81.5
Bleeding	167	41.9
Rash	146	36.6
Headache	88	22.1
Muscular pain	87	21.8
Nausea/Vomiting	79	19.8
Other	110	27.8
Don't know	38	9.5
**Medicines against Dengue** Φ		
Antibiotics	22	5.6
Antimalarials	19	4.9
Antipyretics	89	22.8
Pain killers	34	8.7
Don't know	263	67.3
Others	21	5.4

* Rest of the interview was continued with only those respondents who replied ‘yes’ to this question.

** Only those respondents are included in this question who considered Dengue to be transmissible.

Φ Multiple response options.

Data showing the extent of knowledge regarding vector breeding sites, mosquito bite time and preventive practices is presented in Table 3. About one-half (51.1%) of those interrogated were cognizant of the fact that the dengue mosquito breeds in clean standing water. Most of the people knew that the mosquito usually bites either at sunset/dusk (57.5%) or at sunrise/dawn (44%). Mosquito sprays and mosquito mats were considered the most common choices for prevention, stated by 54.9% and 50.1% of the sample, respectively.

**Table 3:** Knowledge of Vector Characterisitcs and Preventive Measures.

Variable	n	%
**Common breeding site** Φ		
Standing Clean Water	204	51.1
Garbage/Trash	165	41.4
Standing Dirty Water	158	39.6
Plants/Vegetation	33	8.3
Running Clean Water	27	6.8
Running Dirty Water	22	5.5
Others	26	6.5
Don't know	19	4.8
**Most frequent mosquito bite time** Φ		
Sunset/Dusk	230	57.5
Sunrise/Dawn	176	44.0
Morning	114	28.5
Night	97	24.3
Noon	40	10.0
Don't know	21	5.3
**Knowledge of Preventive Measures** Φ		
***Prevention against bite of mosquito***	**978**	**76.3** *
Mosquito Spray	219	54.9
Mosquito Mat/Coil/Liquid Vaporizer	200	50.1
Window & Door Screen	108	27.1
Mosquito Net	103	25.8
Cleaning House	90	22.6
Mosquito Repellant/Cream	88	22.1
Cleaning of garbage/trash	87	21.8
Covering of body with clothes	42	10.5
Use of Fan	27	6.8
Use of Smoke to drive away mosquitoes	13	3.3
Electrocutor	1	0.3
***Eradication of breeding sites of mosquito***	**220**	**17.2** *
Prevent Water Stagnation	108	27.1
Covering containers	69	17.3
Changing water in storage tanks	43	10.8
Cutting trees/vegetations	23	5.8
Others	60	15.1
Don't know	17	4.3

* Percentage of the total responses falling in this category.

Φ Multiple Response.

Preventive practices regarding dengue were consistent with the knowledge about these practices, with majority of the respondents relying on mosquito sprays (48%) and/or mosquito mats and vaporizers. Table 4 lists the distribution of preventive practices in our sample.

**Table 4:** Common Preventive Practices Against Dengue.

Preventive PracticesΦ	n	%
Mosquito Spray	180	48.1
Mosquito Mat/Coil/Liquid Vaporizer	176	47.1
Window & Door Screens	101	27.0
Prevent Water Stagnation	78	20.9
Cleaning house	72	19.3
Cleaning of garbage/trash	54	14.4
Covering Container	49	13.1
Mosquito Repellant/Cream	39	10.4
Covering body with clothes	25	6.7
Changing water in storage tanks	23	6.1
Use of Fan	16	4.3
Cutting trees/vegetations	12	3.2
Use of Smoke to drive away mosquitoes	8	2.1
Mosquito Net	7	1.9
Others	40	10.6
None	32	8.6

Φ Multiple Response variables.

Sufficient knowledge about dengue was found to be in 38.5% of the sample, with 66% of these in Aga Khan University Hospital and 33% in Civil Hospital Karachi. Table 5 shows the association between knowledge scores and factors that may affect knowledge in the population. Significant associations were found between knowledge scores and education (<0.001), income (<0.001), and the hospital of interview (<0.001). Respondents with education up to secondary school (grade-6) were more ignorant as compared to those who had completed ten years of schooling (48.4% versus 29.7%). Prevalence of insufficient knowledge was greater amongst respondents from Civil Hospital compared to those from AKUH (60% versus 40%).

**Table 5:** Association between Knowledge Scores and Determining Factors.

Variables	Insufficient knowledge		Sufficient Knowledge	
Age (years) (p = 0.166)	n	%	n	%
18–25 (102)	83	30.2	38	22.1
26–40 (193)	126	45.8	85	49.4
41–64 (97)	62	22.5	43	25.0
65+ (10)	4	1.5	6	3.5
**Sex (p = 0.164)**				
Male (259)	181	65.8	102	59.3
Female (143)	94	34.2	70	40.7
**Education (p<0.001)**				
Secondary or below (144)	133	48.4	51	29.7
Matric or above (258)	142	51.6	121	70.3
**Income (p<0.001)**				
<3000 (26)	32	12.5	7	4.3
3000–5000 (65)	59	23.0	18	11.0
5000–10000 (100)	74	28.9	38	23.3
10000–20000 (75)	43	16.8	36	22.1
20000–35000 (50)	20	7.8	30	18.4
>35000 (65)	28	10.9	34	20.9
**History of recent fever** * ** (p = 0.409)**				
Yes (73)	45	19.5	28	16.3
No (330)	186	80.5	144	83.7
**Family History of Dengue (p = 0.095)**				
Yes (76)	37	16.1	39	22.7
No (326)	193	83.9	133	77.3
**Hospital (p<0.001)**				
AKUH	110	40.0	115	66.9
Civil	165	60.0	57	33.1

* within the past one week.

Table 6 shows the final multivariate regression model obtained after subjecting ‘age’, ‘income’, ‘education’ and ‘hospital’ to multiple logistic regression against knowledge as the dependent variable. Even though ‘age’ did not attain significance in the univariate analysis, we used it in the final model on the basis of its biological significance. After adjusting for confounding, income and hospital-presented-to were found to be two parameters that were independent predictors of knowledge in our population. Those earning Rs. 20,000–35,000 (US$ 300–550) monthly were found to be 3.9 times more likely to possess sufficient knowledge, compared to those earning less than Rs. 3,000 (US$ 500) per month. Furthermore, people presenting to AKUH were found to have greater probability of having ‘sufficient knowledge’ (OR = 2.1).

**Table 6:** Multiple Regression Model; Independent Predictors and Adjusted Odds of Factors Determining Knowledge.

	Insufficient Knowledge		Sufficient Knowledge		Adjusted OR
	n	%	n	%	
**Total (447)**	275	61.5	172	38.5	
**Income (p = 0.05)**					
<3000	32	12.5	7	4.3	**1**
3000–5000	59	23.0	18	11.0	**1.27**
5000–10000	74	28.9	38	23.3	**2.05**
10000–20000	43	16.8	36	22.1	**2.42**
20000–35000	20	7.8	30	18.4	**3.91**
>35000	28	10.9	34	20.9	**3.2**

**Figure 1:**
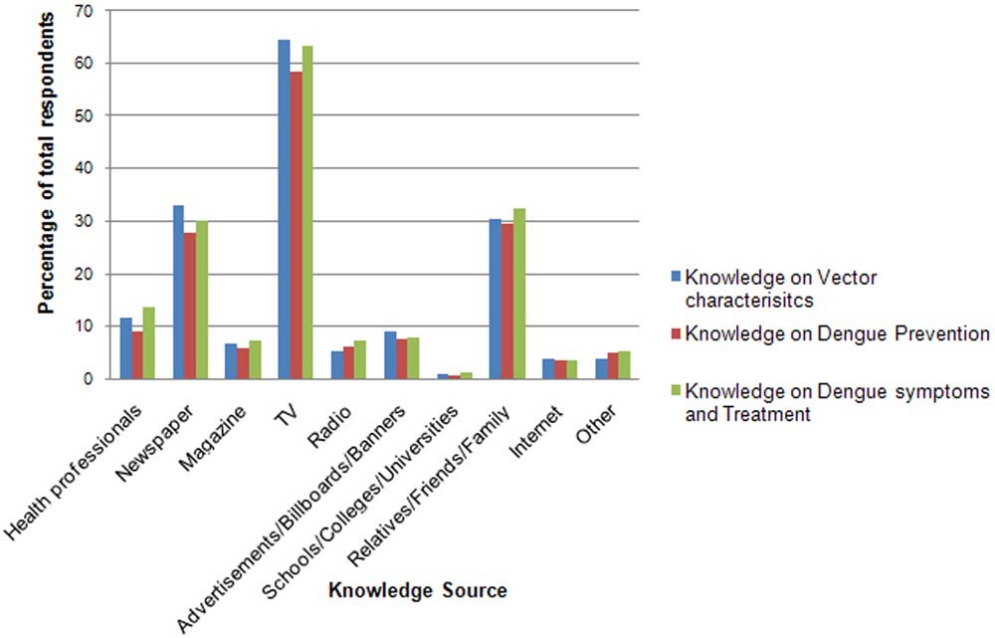
Sources of Information on Various Aspects of Dengue Fever.

Figure 1 is a bar chart showing a comparison between the various sources of information for knowledge on dengue spread, its prevention, symptoms and treatment. Television was identified as the major source of public information in all three aspects of knowledge with similar proportions of respondents (an average of 62%) in each category. Friends/relatives were identified as the second most frequent source (an average of 31% in each category). Newspapers were considered as a source of information by only about one-third of respondents in all categories.

## Discussion

Both hospitals used in our study were large, tertiary-care health facilities. However, Civil Hospital Karachi (CHK), being a public hospital, draws a significantly greater number of patients compared to AKUH because it is more affordable. Samples from both hospitals showed some similarities in the basic demographics. Education level for the total sample did not reflect the national average since a significant proportion (∼80%) of Pakistani population is considered illiterate [12], [13]. However, sample from CHK comprised mostly of illiterate people, and overall the sample could be considered representative for Karachi.

It was observed that all of the respondents who had never heard of the word “dengue” before belonged to the sample from Civil Hospital. Even though many respondents were familiar with dengue being a communicable disease which spreads via mosquito vector, yet several misconceptions were identified. According to WHO guidelines on dengue [6], the *Aedes aegypti* mosquito typically bites during the day. A considerable proportion of respondents regarded Anopheles mosquito (malarial vector) and *Aedes Aegypti* to have similar characteristics and habitat, along with their transmission patterns. This is most likely due to high prevalence of malaria causing Anopheles mosquito in Pakistan, the knowledge about which is generalized to the dengue mosquito by the common person. Despite the fact that majority of the people had heard about dengue somewhere, a good proportion did possess deficiencies in their knowledge about the disease. A large number of people considered dengue to be contagious, and an almost equal number were not sure whether it has person-to-person transmission. These findings are consistent with similar studies done in the South Asian region [14], [15].

Three manifestations of dengue are currently known; dengue fever, dengue hemorrhagic fever and dengue shock syndrome. However, fever is the most common presenting symptom in all of them [16]. Our sample showed considerably good knowledge about the symptoms, with fever being correctly accounted as the most common. Adequate knowledge on dengue symptoms has been reported in similar studies done in India and Brazil [14], [17]. Bleeding and rashes were some of the other common symptoms mentioned. As reflected by Benthem et al. in their study carried out in Northern Thailand , rash or bleeding is a specific symptom of dengue infection and not common in other febrile illnesses indicating that the majority of people can distinguish dengue infection from other diseases [6], [18]. These responses showed that the awareness of symptoms was good. Based on these findings, one could propose that dissemination of knowledge about symptoms was sufficient and effective. Knowledge about the treatment of dengue was not prevalent. Although a number of people did identify anti-pyretics as being important, the majority were unaware.

Preventive measures preferred were use of mosquito sprays and coils. Several studies have reported these methods to be most effective means of prevention [19], [20]. Measures aimed at preventing water stagnation, which serves as local breeding sites were the second most popular techniques in use. This is in accordance with studies done in Thailand which reported a significant reduction of dengue vectors and dengue hemorrhagic fever cases in areas having clean-up campaigns before and during rainy seasons [18].

Window and door screens were also a popular method of vector control. Window curtains and domestic water container covers treated with insecticide can reduce densities of dengue vectors to low levels and potentially affect dengue transmission [21]. These results displayed that the study population was using adequate preventive methods aimed at controlling both the vector's breeding and its spread.

We asked individuals about their source of information on dengue. Since city administration had never enacted any billboards on dengue, prior to the recent outbreaks; neither were there any mass awareness campaigns or steps taken to spread information for the general public on dengue, all sources disseminating knowledge about the disease were a recent reaction of government and public to the current outbreak. We divided the knowledge obtained into three categories. However, knowledge on all aspects seemed to be closely linked, as the frequencies recorded were almost equal for knowledge on prevention, vector characteristics, symptoms and treatment of dengue. Therefore, irrespective of the source of information, a comprehensive knowledge of the disease was being provided. Most important role seemed to be played by media including television and newspapers. Since the number of local television channels had increased relaying information in the national language in the country, television viewership had increased among the masses, and this may be the reason why it was considered the source of information by the majority. The role of newspapers was also important, even though this may not be a representative due to the better level of education observed in our sample, which is inconsistent with the country's population.

Assessing specific demographic factors for association with the knowledge did lead to particular trends. Level of education, for one, determined the knowledge of scores, whereby our dichotomous category revealed that people who had received at least one certificate of education had significantly better knowledge. Furthermore, a decreasing prevalence of poor knowledge was seen as income increased. Another factor that seemed to determine knowledge in our sample was a family history of dengue. Insufficient knowledge was found to be more in the group where no person in the family had previously been exposed to dengue. We can thus assume that drift of information occurs within a family, and that knowledge seeking behavior also improves in such families. As dengue had been a cause of concern in Karachi at the time of our interviews , and since media was giving enough air-time to this particular disease, we had assumed a high prevalence of sufficient knowledge in our sample [9].

Several studies have shown that a higher socio-economic status (SES) correlates with better knowledge scores [22]–[24]. Our finding points to the fact that the people in a higher SES may have factors other than a better education influencing their awareness about the particular subject. For example, a well informed neighborhood, daily correspondence with people more aware on the subject, better and easier access to information (internet facilities, television, newspaper), and an indirect effect of education reflected by the ability to understand and comprehend information. This suggests that the means to disseminate knowledge regarding dengue among the general population has been more beneficial for those with a better income, and there has been a smooth transition of increasing knowledge with each ascending tier of income. Comparatively, those earning less than ten thousand rupees per month had a prevalence of sufficient knowledge of only 37.6%, while they represented more than half of our sample. These findings suggest that more effective programs for population awareness need to be implemented, which would target population in lower SES groups.

The above observations may be true only for the study population because of convenience sample and cannot be generalized to other populations belonging to different socio-economic or cultural backgrounds. Local studies are needed to provide the true picture about awareness regarding dengue fever so that appropriate specific action can be taken for control of disease.

The questionnaire, though pre-tested, was not validated. Demographic data such as locality within Karachi were not analyzed in the questionnaire. Some localities have had an outbreak of dengue so it can be hypothesized that people from those localities have a higher knowledge but we did not assess this factor. The aim of the study was to evaluate the knowledge and perception regarding dengue and finding an association with specific locality was not part of our objectives.

### Conclusion

We have found a low prevalence of sufficient knowledge in our sample population based on overall knowledge score on dengue. However, isolated knowledge on symptoms and prevention is adequate; with preventive measures mainly focused towards protection from mosquito bites. The available evidence from Pakistani population is limited and there is a need for a nationally representative survey to assess the knowledge and attitudes regarding dengue and any misconception in the general population.
